# Time-resolved phosphoproteome and proteome analysis reveals kinase signaling on master transcription factors during myogenesis

**DOI:** 10.1016/j.isci.2022.104489

**Published:** 2022-05-30

**Authors:** Di Xiao, Marissa Caldow, Hani Jieun Kim, Ronnie Blazev, Rene Koopman, Deborah Manandi, Benjamin L. Parker, Pengyi Yang

**Affiliations:** 1 Computational Systems Biology Group, Children’s Medical Research Institute, Faculty of Medicine and Health, The University of Sydney, Westmead, NSW 2145, Australia; 2 Charles Perkins Centre, School of Mathematics and Statistics, The University of Sydney, Sydney, NSW 2006, Australia; 3 Centre for Muscle Research, Department of Anatomy and Physiology, School of Biomedical Sciences, The University of Melbourne, Melbourne, VIC 3010, Australia

**Keywords:** cell biology, Developmental biology, proteomics

## Abstract

Myogenesis is governed by signaling networks that are tightly regulated in a time-dependent manner. Although different protein kinases have been identified, knowledge of the global signaling networks and their downstream substrates during myogenesis remains incomplete. Here, we map the myogenic differentiation of C2C12 cells using phosphoproteomics and proteomics. From these data, we infer global kinase activity and predict the substrates that are involved in myogenesis. We found that multiple mitogen-activated protein kinases (MAPKs) mark the initial wave of signaling cascades. Further phosphoproteomic and proteomic profiling with MAPK1/3 and MAPK8/9 specific inhibitions unveil their shared and distinctive roles in myogenesis. Lastly, we identified and validated the transcription factor nuclear factor 1 X-type (NFIX) as a novel MAPK1/3 substrate and demonstrated the functional impact of NFIX phosphorylation on myogenesis. Altogether, these data characterize the dynamics, interactions, and downstream control of kinase signaling networks during myogenesis on a global scale.

## Highlights

• Phosphoproteomic and proteomic maps of myogenic differentiation of C2C12 cells
• Myogenic kinome activity and kinase-substrates prediction using machine learning
• MAPK1/3 and MAPK8/9 inhibition unveil shared and distinctive effects on myogenesis
• Validation of NFIX phosphorylation by MAPK1/3 and its impact on myogenesis

## Graphical abstract

**Figure:**
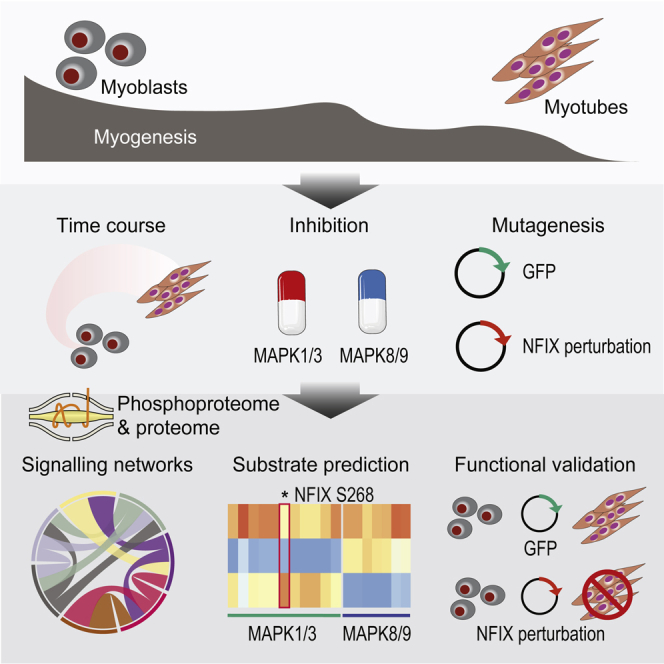

## Introduction

The bulk of skeletal muscle is composed of post-mitotic multinucleated myofibers that form via the fusion of mono-nucleated progenitor myoblasts (Dittmar and Zänker, 2011). Myofiber formation is achieved via *myogenesis*, a highly ordered process that includes proliferation, differentiation, and ultimately cell fusion (Knight and Kothary, 2011). At the molecular level, the specification of myogenic lineage and the differentiation of multinucleated myotubes are orchestrated by signaling cascades and the underlying transcriptional regulation (Chal and Pourquié, 2017). In the last few decades, much of the effort has been devoted to investigating the roles of kinases in regulating myogenesis using various biochemical techniques (Knight and Kothary, 2011). Although these studies have led to the identification and functional characterization of various individual kinases in regulating different phases of myogenesis, the low-throughput nature of the employed techniques impedes the reconstruction of global signaling cascades, the characterization of their dynamics across the kinome, and the identification of their substrates involved in myogenesis. In parallel biochemical perturbation analysis of kinases, an extensive body of work on the transcriptome during myogenesis has identified various master transcription factors (TFs) that undergo temporal expression changes during myogenic differentiation and cooperatively establish and modulate the final transcriptional program (Asfour et al., 2018; Hernández-Hernández et al., 2017). Nevertheless, the *trans*-regulatory networks from which the global signaling cascades culminate in the activation of the underlying transcriptional regulation in myogenic differentiation remain largely unexplored.

To investigate the global dynamics of signaling and the underlying transcriptional regulation on modulating myogenesis, here we profiled the time-resolved proteome and phosphoproteome during a 5-day C2C12 myogenic differentiation from myoblasts to myotubes. The temporal phosphoproteomics provided the opportunity to characterize the kinome activities and crosstalk through signaling networks. Specifically, we found that multiple mitogen-activated protein kinases (MAPKs) showed significant changes in activities during the early stages of myogenic induction. This is in agreement with previous reports that various MAPKs play critical roles in myogenesis (Perdiguero et al., 2007; Wu et al., 2000; Xie et al., 2018). However, the investigation of downstream substrates was complicated owing to the similarity in the substrate temporal phosphorylation profiles and the kinase recognition motifs among MAPKs. To dissect the roles of different MAPKs in regulating myogenesis, we profiled the phosphoproteomics and the proteomes of differentiated cells treated with the MAPK1/3 and MAPK8/9 specific inhibitors and compared those to the controls. By integrative analysis of the inhibition profiles, we were able to identify the shared and unique pathways regulated by MAPK1/3 and MAPK8/9. In addition, the integration of the inhibition phosphoproteomic data allowed us to dissect kinase substrates that are specific to MAPK1/3 and MAPK8/9 phosphorylation. Next, we experimentally validated nuclear factor 1 X-type (NFIX), a master TF known to be involved in myogenesis (Rossi et al., 2016), as a substrate phosphorylated by MAPK1/3 at serine 268 (S286) as predicted by our computational framework. Overexpression of either wild-type (WT) NFIX or a phospho-mutant of NFIX in C2C12 cells identifies its functional impact on myogenesis. Further proteomic profiling of cells over-expressed with NFIX-WT and NFIX-mutant reveals downstream targets of these *trans*-regulatory networks. Together, the integrative view of the dynamics in signaling and protein abundance and their disruption by kinase inhibition and NFIX mutagenesis provides new insight into signaling cascades and their downstream *trans*-regulatory networks that underlie myogenesis.

## Results

### Time-dependent phosphoproteomic and proteomic profiling of C2C12 cells during myogenic differentiation

**Figure 1:**
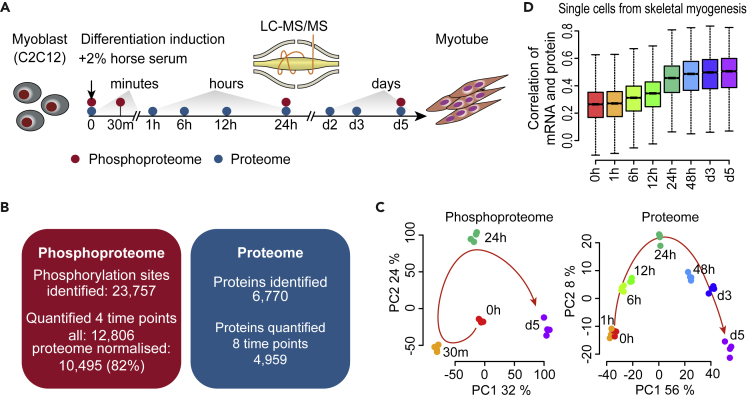
Global profiling of the phosphoproteome and proteome during myogenic differentiation of C2C12 murine myoblasts (A) Schematic summary of myogenic differentiation of C2C12 murine myoblasts to myotubes. Phosphoproteome and proteome were profiled at indicated time points from the differentiation induction of myoblasts to the formation of myotubes. (B) Summary statistics of phosphosites and proteins identified and quantified in the myogenic differentiation experiment. (C) Principal component analysis (PCA) summarizing temporal dynamics of global changes in the phosphoproteome and proteome during myogenesis. Each point represents data collected at the indicated time point during differentiation. The color of each circle denotes a time point during differentiation. (D) Correlation of the time-course proteome with the mRNA of single cells isolated from *in vivo* skeletal myogenesis during mouse development (Cao et al., 2019). See also Figure S1.

To map the dynamics of signaling and downstream regulation during myogenesis, we profiled the phosphoproteome and proteome of C2C12 myoblasts and across their differentiation to myotubes during the five-day myogenic induction (Figure 1A). In total, we identified 23,757 phosphorylation sites, of which 12,806 were quantified across the four profiled time points (0, 30m, 24h, and d5), and 10,495 were further normalized by the total proteome (Figure 1B and Table S1). On the proteome level, we identified 6,770 proteins within which 4,959 were quantified across the eight profiled time points (0, 1, 6, 12, 24h, d2, d3, and d5) (Table S2). Up-regulation of key genes associated with myogenesis confirmed successful induction of the myogenic program including MYH1/3 (Schiaffino et al., 2015), ACTA1 (Sin et al., 2016), and MYOM1 (Obermann et al., 1997) (Figure S1A), and the global hierarchical clustering of the phosphoproteome (Figure S1B) and proteome (Figure S1C) indicate high-quality and reproducibility of the data.

Principal component analysis (PCA) (Figure 1C) demonstrated a clear time-dependent separation of both phosphoproteomics and proteomics data. In particular, while there is a clear change in the global phosphorylation at 30 min compared to 0, the changes in the proteome level only become apparent at 6 h, highlighting the fast response in phospho-signalling and the additional time required for the signaling cues to be converted to and reflected on changes in protein abundance in response to differentiation induction. To further quantify the proteome dynamics, we calculated the correlation of fold change (compared to 0) of each of all times (Figure S1D) and fitted a locally estimated scatterplot smoothing (loess) curve to the correlations from any two adjacent time points (Figure S1E). These data suggest significant proteome changes from across the profiled time points until day 3, marked by the plateau onwards to day 5. Consistent with this, we correlated our time-course proteome data with the mRNA of single cells isolated from *in vivo* skeletal myogenesis during mouse development (E9.5 to E13.5) (Cao et al., 2019) (Figure 1D) and found a clear increase in concordance of the two omics layers at 12–24 h and plateau at day 3.

### Kinase activity inference and kinase-substrate prediction provide a global map of signaling cascades during myogenesis

**Figure 2:**
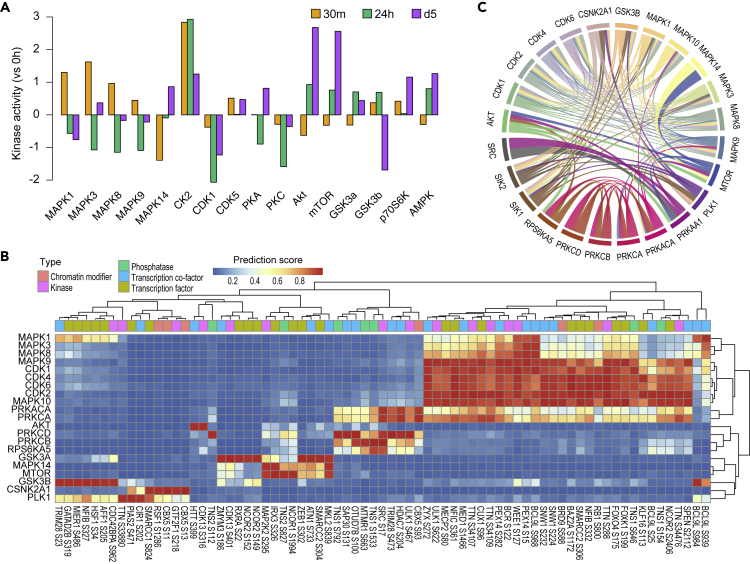
Inference of signaling activity and prediction of substrates for key kinases involved in myogenesis (A) Inference of kinase activity using KinasePA (Yang et al., 2016) at 30 min, 24 h, and day 5 (relative to 0) of the phosphoproteome during myogenesis. (B) Heatmap showing selected kinases and their predicted substrates from the time-dependent phosphoproteomic data using PhosR (Kim et al., 2021a) (see STAR Methods). Kinase substrates are filtered and color coded for those that are kinases, phosphatase, transcription factors, transcription co-factors, and chromatin modifier. (C) Signalome networks inferred from predicted kinase-substrate relationships of kinases. The width of edge between kinases represents the amount of shared substrates indicating the degree of their co-regulation. See also Figure S2.

Given the critical roles played by kinases in driving the activation, proliferation, differentiation, and fusion in myogenesis (Knight and Kothary, 2011), we first characterized the activity of kinases based on the phosphorylation level of their known substrates from the phosphoproteomics data at the profiled time points. Our analysis revealed that MAPK signaling, marked by MAPK1/ERK2, MAPK3/ERK1, MAPK8/JNK1, and MAPK9/JNK2, were among the first activated cascades at the onset of myogenic induction (Figure 2A). Although these MAPKs show strong activation at the 30m time point (compared to 0h myoblasts), their activity underwent a significant reduction at 24h post myogenic induction, confirming a time-dependent activation and inhibition of the MAPK signaling during myogenesis (Adi et al., 2002). Unlike MAPK1/3/8/9 and consistent with previous studies (Cabane et al., 2003), the activity of MAPK14/p38α was induced at the later stage of myogenic differentiation. Besides MAPK signaling, the casein kinase 2 (CK2) also appears to be activated early during the differentiation but its activity continued throughout the differentiation process, suggesting that its activity is required across different phases in establishing the myogenic program (Salizzato et al., 2019). Previous studies have identified cyclin-dependent kinases (CDKs) as the key regulators during myogenesis (Skapek et al., 1995). We found that the activity of CDK1 reduced significantly post 24h, whereas CDK5 showed a mild increase in activity at both early and late time points. Both protein kinase A (PKA) and C (PKC) have been demonstrated to play important roles in regulating myogenesis (Chen et al., 2005; Goel and Dey, 2002). In particular, the down-regulation of PKC has been implicated to activate MAPK8/9 and promote myogenic differentiation. In agreement with these results, we observed a reduction in PKC activity at the onset of differentiation which extended beyond 24 h post-induction. Also of interest is the activation of Akt and mammalian target of rapamycin (mTOR) signaling cascades at 24 h and d5. Glycogen synthase kinase 3β (GSK3β) is a known substrate of Akt and is negatively regulated by Akt activity. The activation of Akt at 5d closely mirrors the deactivation of the GSK3β activity and is consistent with the previous report that the inhibition of GSK3β stimulates myogenic differentiation (Goel and Dey, 2002). In contrast, ribosomal S6 kinase (S6K) is a known substrate of mTOR, a key regulator for the terminal differentiation of C2C12 myoblasts (Shu and Houghton, 2009), and is positively regulated by its activation. The activation of S6K at d5 is in agreement with its relationship with mTOR and is consistent with the previous findings linking it to hypertrophy (Cuenda and Cohen, 1999). Finally, we found the activity of AMP-activated protein kinase (AMPK) increased at 24 h and d5. Interestingly, previous reports suggest a negative effect of 5-aminoimidazole-4-carboxamide 1-β-D-ribonucleoside (AICAR)-induced AMPK phosphorylation on myogenesis (Williamson et al., 2009). Together, these data provide a time-dependent global view of kinase activity during myogenesis.

While most of the kinases detected above (Figure 2A) have been reported for their role in regulating myogenesis, the substrates that each kinase phosphorylates and the dynamics of these substrates during the differentiation process remain largely uncharacterized. To this end, we computationally predicted kinase-specific substrates by modeling from both the kinase recognition motifs and the phosphorylation profiles of known substrates of each kinase using PhosR package (Kim et al., 2021a). The utility of the phosphorylation dynamics from global phosphoproteomics data is especially important for predicting kinase substrates that are context-relevant (Yang et al., 2016) and here, enabled us to identify novel putative substrates of kinases that are involved in the myogenic differentiation. Among these putative substrates, many of them are kinases and downstream transcriptional regulators such as TFs, co-factors, and chromatin modifiers (Figure 2B), revealing cascades of signal transduction from phosphorylation to downstream transcriptional and epigenetic regulation. By utilizing the kinase-substrate prediction results, we reconstructed signalome networks based on the shared substrate proteins of kinases (Figure 2C). This reconstructed signalome revealed a widespread crosstalk among key kinases of myogenesis, especially within and among MAPKs and CDKs. These data highlight the extensive interconnection among signaling networks to make critical cell-fate decisions and prioritize a large number of novel substrates that may play critical roles at different phases (e.g., proliferation, differentiation, and fusion) in controlling myogenic progression.

### Proteomic profiles of kinases and gene pathways largely mirror their signaling activity

During myogenesis, the signaling cascades culminate in the activation and rewiring of transcriptional networks which guide the cells toward the generation of multinucleated myofibers. The change in the transcription program during myogenic progression is ultimately reflected on the proteome level. By measuring global proteomes during the differentiation process, we found that the protein abundance of kinases largely mirrors their signaling activity (Figure S2A). For example, in concordant with their reduced kinase activity (Figure 2A), the protein levels of MAPK1, MAPK3, and CDK1 decreased during the differentiation. In contrast, AKT2 and MTOR protein levels increased as did their kinase activity. A clear exception is MAPK14 which appears to have a reduction in protein level but an increase in kinase activity. We next partitioned the global proteomics data into distinctive temporal clusters (Figure S2B) from which we detected enriched gene pathways using ClueR (Yang et al., 2015) and Reactome database (Fabregat et al., 2018) (Figure S2C). By ordering the clusters based on their temporal profiles using the Minardo package (Kaur et al., 2020), we found the nephrin interactions/cell-cell communication pathways were up-regulated first roughly at 1h post differentiation induction (Figure S2D). This was followed by the down-regulation of the cell cycle mitotic pathway at around 12h and the up-regulation of the muscle contraction pathway shortly after. The glucose metabolism/respiratory electron transport pathway was up-regulated after 24h and was closely followed by the down-regulation of the mitotic G1/S transition. Finally, the gene pathway that is activated by peroxisome proliferator-activated receptor α (PPARα) was turned off around 48h. As PPARs are associated with adipogenesis (Brun et al., 1996) and can transdifferentiate myoblasts to mature adipocytes (Hu et al., 1995), the down-regulation of PPARα targeted genes at 48h suggests a commitment to myogenic lineage. Together, these time-dependent changes in gene expression pathways, as reflected by their protein abundance, mirror the signaling activity such as the reduction of CDK1 activity and increase of Akt and mTOR activity and mark a coordinated regulation of transcriptional and translation programs in response to signaling cascades.

### Kinase-specific inhibitions of MAPK1/3 and MAPK8/9 reveal shared and distinctive effects on myogenesis

**Figure 3:**
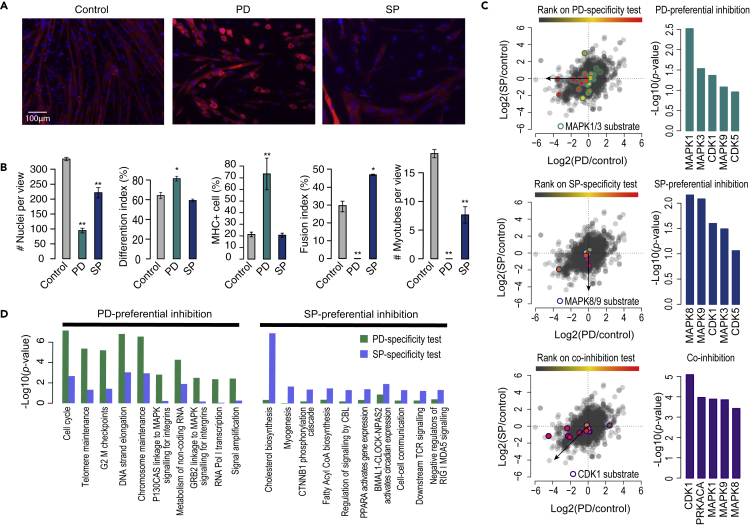
Dissection of MAPK1/3 and MAPK8/9 specific effects on C2C12 myogenesis (A) Immunofluorescence microscopy of differentiated C2C12 at day 3 where cells were either from normal differentiation protocol (control), or treated with MAPK1/3 inhibitor PD0325901 (PD) or MAPK8/9 inhibitor SP600125 (SP) at the onset of myogenic induction. Cells were stained for DAPI (blue) and MHC (red), a myogenic marker. (B) Nuclei, MHC + cells, and myotubes were counted based on staining described in (A). Differentiation index, the percentage of MHC + cells, and fusion index were calculated based on the counting of nuclei, MHC + cells and myotubes (See STAR Methods). Data are presented as mean ± SD for n = 3. p-values were computing using t-test comparing PD or SP to control. ∗p < 0.05, ∗∗p < 0.01. (C) Direction-based integrative analysis of the phosphoproteome from the inhibitor experiment using KinasePA (Yang et al., 2016) for kinases preferentially inhibited by PD (top), SP (middle), and both (bottom). In the scatterplots, known substrates of MAPK1/3 (top), MAPK8/9 (middle), and CDK1 (bottom) are highlighted and colored from high (red) to low (gray) based on their significance in the tested directions indicated by the arrows. The bar plots beside the three scatterplots show the most inhibited kinases in the tested directions. (D) Direction-based integrative analysis of the proteome from the inhibitor experiment using directPA (Yang et al., 2014) for pathways (from the Reactome database) preferentially inhibited by PD and SP. Select of significantly inhibited pathways by PD and/or SP are shown. Scale bar, 100 μm (A). See also Figure S3.

Although the time-dependent phosphoproteomic data unveil the initial up-regulation in MAPK1/3/8/9 activity, the specific roles played by each MAPK at different phases of the myogenic progression and their downstream pathways remain to be characterized. To answer this, we treated myoblasts with either the MAPK1/3 inhibitor PD0325901 (PD) or the MAPK8/9 inhibitor SP600125 (SP) prior to the induction of differentiation to examine the specific effects of MAPK1/3 and MAPK8/9 on the myogenic program (Figure S3A). Compared to the vehicle-treated control cells on day 3 after myogenic induction, both PD and SP caused morphological changes where cells remained round shaped and mononucleated with PD treatment or elongated with many more nuclei fused into thicker myotubes with SP treatment (Figure 3A). MAPK1/3 inhibition led to a significant reduction in the number of myoblasts marked by the low number of nuclei compared to the controls (Figure 3B, first panel) and is in agreement with the role of MAPK1/3 in inducing myoblast proliferation (Kook et al., 2008). The increased differentiation index and the high percentage of myosin heavy chain positive (MHC+) cells (Figure 3B, second and third panels) suggest that the inhibition of MAPK1/3 promotes myoblasts differentiation to myocytes and is consistent with its inhibitory role in the differentiation phase (Bennett and Tonks, 1997). As these myocytes also fail to form myotubes, this suggests that MAPK1/3 inhibition prevents myocytes from becoming fully matured and capable of myogenic fusion. In comparison, cells treated with SP showed similar differentiation capacity compared to controls but, interestingly, increased fusion, leading to lower number of myotubes each with the fusion of more nuclei (Figure 3B).

To investigate the changes in the signaling cascades and downstream translational regulation, we next profiled the phosphoproteome and the proteome of PD and SP treated cells (Figure S3A, Tables S3 and S4). The reduction in phosphorylation levels of MAPK1/3 substrates (STMN1 S25 and AHNAK S4890) and MAPK8/9 substrates (JUN S63 and JUN S73) demonstrates the effectiveness of the inhibition experiments (Figure S3B), and the reproducibility of the biological replicates indicates the high quality of both the phosphoproteomic and the proteomic data (Figures S3C and S3D). Through comparative analysis of the phosphoproteomics of PD and SP treated cells, we found, as expected, that PD and SP preferentially inhibit MAPK1/3 and MAPK8/9, respectively, and both led to the inhibition of CDK1 (Figure 3C and Table S5). Comparative analysis of the proteome confirms that the cell cycle is inhibited by both inhibitors but significantly more so by PD treatment (Figure 3D). Nevertheless, the PD and SP treatments do show distinctive effects on molecular pathways during myogenic differentiation. In particular, while PD treatment led to a stronger reduction in the cell cycle, SP treatment appears to specifically target pathways related to cholesterol biosynthesis and myogenesis (Figure 3D). These findings agree with the results from image analysis where PD and, to a lesser degree, SP led to a significant reduction in cell proliferation presumably owing to the existence of the cell cycle triggered by the inhibition of CDK1.

### Identification and validation of NFIX as a novel MAPK1/3 substrate in regulating myogenesis via phosphorylation

**Figure 4:**
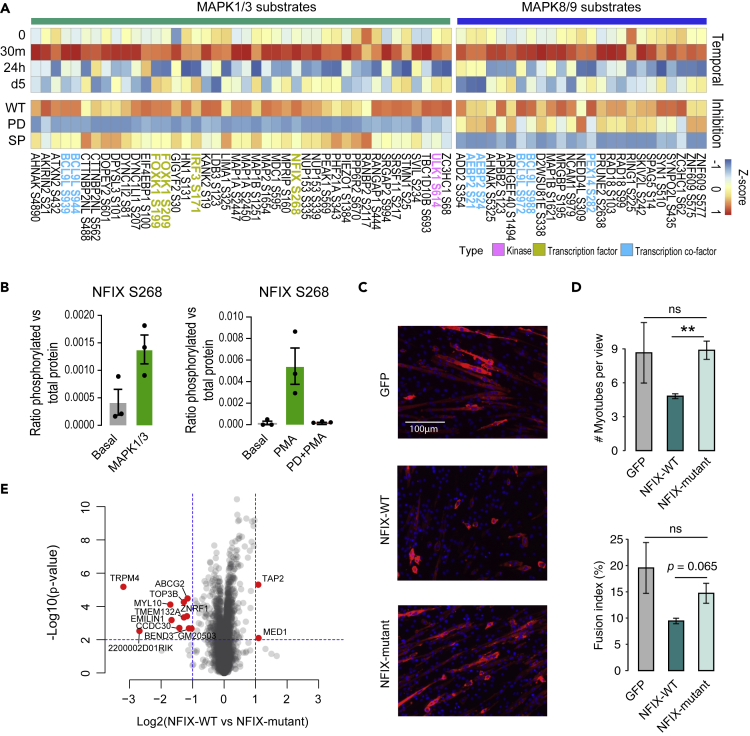
Prediction and validation of MAPK1/3 and MAPK8/9 substrates and reconstruction of signaling network dynamics during myogenesis (A) Heatmap showing the temporal (up panel) and inhibition (down panel) phosphorylation profiles (*Z* score transformed) of predicted substrates of MAPK1/3 and MAPK8/9. TFs, co-factors, and kinases in the list are highlighted. (B) Validation of NFIX S268 as a novel MAPK1/3 substrate. Left panel showing the quantification of NFIX S268 phosphorylation in basal and MAPK1/3 treatment using *in vitro* kinase assay. Right panel showing the quantification of NFIX S268 phosphorylation in C2C12 cells in basal condition or treated with PMA (a MAPK1/3 activator) or the combination of PD and PMA. (C) Immunofluorescence of differentiated C2C12 at day-3 with overexpression of either GFP, wild-type (WT) NFIX, or a phospho-dead mutant of NFIX prior to the myogenic induction. Cells were stained for DAPI (blue) and MHC (red). (D) Quantification of morphological changes in overexpression experiments described in (B). Myotubes were counted based on DAPI and MHC staining. Fusion index was calculated by dividing the number of nuclei within multinucleated myotubes by the total number of nuclei. Data are presented as mean ± SD ∗p < 0.05, ∗∗p < 0.01, ns, no significance. (E) Volcano plot of NFIX-WT overexpression compared to NFIX-mutant overexpression with both normalized to GFP overexpression. The significant differentially expressed (DE) proteins are highlighted in red. See also Figure S4.

By combining the temporal myogenesis and the inhibitor-specific phosphoproteomic data, we further refined computational predictions to identify substrates that are specific to MAPK1/3 or MAPK8/9. Although the temporal profiles and kinase motifs of potential substrates for these two sets of kinases closely resemble each other and therefore may not be sufficient to resolve their specificity, the inhibition profiles clearly separate them into two distinctive groups and thus enabled us to precisely dissect the candidates into those that are specific to MAPK1/3 and MAPK8/9 (Figures 4A and S4A). Within the putative substrates, several of them are TFs (Figure S4B) and co-factors that may serve as keys to convert upstream signaling to downstream transcriptional regulation. Among the TFs, the NFIX has been reported as a master regulator for the temporal progression of muscle generation in mice (Rossi et al., 2016), yet to our knowledge, it is unknown if phosphorylation regulates its function. Our data identified the phosphorylation of NFIX S286 as a top predicted substrate of MAPK1/3 which lies within the CTF/NF-I DNA-binding domain. To validate this putative kinase-substrate relationship we immunoprecipitated FLAG-tagged NFIX and performed an *in vitro* kinase assay with MAPK3 which led to an increase in phosphorylation on NFIX S286 compared to basal (Figure 4B). Next, we pharmacologically activated MAPK1/3 with phorbol 12-myristate 13-acetate (PMA) with or without the presence of PD inhibition and quantified phosphorylation following immunoprecipitation (Meier et al., 1991) (Figure 4B). We observed an increase in phosphorylation on NFIX S286 in PMA treated cells compared to basal and this phosphorylation was abolished by the addition of PD. These data support that NFIX S286 is a novel substrate of MAPK1/3.

To evaluate the functional impact of this phosphorylation on myogenesis, we next over-expressed either GFP, WT NFIX, or a phospho-dead mutant of NFIX in which eight serine residues surrounding S286 were mutated to alanine (S265/267/268/271/272/273/274/275A) in C2C12 cells prior to the induction of myogenesis (Figure S4C). This was performed because there was a string of Ser/Thr residues surrounding the proline-directed phosphorylation site of interest. We found that overexpression of NFIX-WT resulted in a decrease in myotube formation relative to the control GFP vector (Figures 4C and 4D). Considering our time-course data indicate that NFIX is progressively down-regulated during myogenesis (Figure S4B), these results suggest that protein abundance of NFIX is tightly regulated during myogenesis, whereby the up-regulation of NFIX in myoblasts disrupts the differentiation process. Notably, the phospho-dead NFIX-mutant rescued this phenotype and showed minimum impact on the myogenic differentiation (Figures 4C and 4D). Proteomic analysis further confirms (Figures S4D, S4E and Table S6), that the NFIX-mutant samples were closely clustered with control GFP than NFIX-WT samples. Differential analysis revealed numerous proteins involved in myogenic regulation, with significantly altered abundance in NFIX-mutant overexpression compared to NFIX-WT overexpression (Figure 4E and Table S7). For example, ABCG2 is a known regulator in myogenesis, whose deletion led to fewer myofibers and delayed muscle regeneration (Doyle et al., 2011). Its depletion in NFIX-WT overexpression compared to NFIX-mutant, therefore, may be a contributor to the reduction in myotube formation. Taken together, these data demonstrate NFIX as a novel substrate of MAPK1/3 and suggest that the phosphorylation of NFIX at S286 is functional and regulates myotube formation in myogenesis.

## Discussion

Myogenic cells must be irreversibly withdrawn from the cell cycle prior to committing to skeletal muscle development (Bentzinger et al., 2012). Cell cycle regulators, such as cyclins, CDKs, are required for the transition from proliferative to differentiation stage by modulating myogenic regulatory factors, such as MyoD and MEF2C (Guo and Walsh, 1997). Although CDK2/4/6 interactions enable progression into successive cell-cycle phases were often reported in the mammalian cell cycle (Malumbres and Barbacid, 2009), we found CDK1/2/4/6 potentially interplay with each other during myogenesis (Figure 2C). Although multiple CDKs and their interactors are required to inhibit myogenic differentiation, our research sheds light on the coordinated regulation of CDKs required for proliferation maintenance. MAPKs may potentially contribute to proliferation through their interconnection with CDKs, which integrate extracellular signals with the cell cycle regulatory system, as MAPK1/3 has been demonstrated to enhance cell cycle progression by assembling and stabilizing cyclin D1-CDK4/6 complexes (Meloche and Pouysségur, 2007). This is further supported by our finding that the inhibition of MAPK1/3 and MAPK8/9 is associated with the CDK1 activity inhibition (Figure 3C). Additionally, the crosstalk within MAPKs may be important in controlling myogenic cell-cycle exit. The absence of MAPK14 resulted in enhanced activation of MAPK8/9, attributing to delayed cell cycle withdrawal (Perdiguero et al., 2007), which is consistent with MAPK14 down-regulation and MAPK8/9 upregulation at an early time point (Figure 2A). Together, our results indicate that interactions within CDKs or MAPKs, or between MAPKs and CDKs at the initial stage are essential for sustaining proliferation, avoiding premature myoblast differentiation.

Besides the cooperative regulation of MAPKs and CDKs at the outset of myogenic commitment, AKT/mTOR signaling cascade appears to be a slower wave following MAPK signaling, as both ATK and mTOR activities were suppressed at the 30 min after induction and progressively increased along with the myogenic differentiation (Figure 2A). Besides NFIX, the other TF predicted as a substrate of MAPK1/3 is FOXK1. It has been shown that FOXK1 represses the expression of MEF2 and FOXO4, and thus promotes myoblast proliferation and suppresses differentiation (Shi et al., 2012). FOXO subfamily members, including FOXO4, are negatively regulated by AKT (Bouchard et al., 2004). This suggests an orderly flow of signaling cascades from MAPK1/3 to AKT, which ensures precise progression of myogenic differentiation. The AKT pathway often signals through its effector mTOR to stimulate protein synthesis in muscle development (Rommel et al., 2001). Additionally, our data showed a significant association of activity between AKT and mTOR (Figure 2C), suggesting that their interconnection plays a role during myogenesis. Together, our results suggest that the time-ordered interplay among key kinases enables the correct progression of successive myogenesis.

Our kinase-specific substrate prediction bridges the gap between signaling cascades and transcriptional regulation as several putative substrates are TFs, TF co-factors (Figure 4A). Among all putative substrates, NFIX and FOXK1 are two TFs that have demonstrable effects on myogenesis. NFIX has a complex role in myogenesis by targeting different important myogenic regulators. Moreover, the duration and magnitude of NFIX expression are vital for controlling the proper timing of myogenic differentiation. NFIX, in particular, can form a complex with PRKCQ for MEF2A activation (Messina et al., 2010), collaborates with SOX6 in repressing MYH7 (Taglietti et al., 2016), and directly represses the myostatin promoter (Rossi et al., 2016). We have shown that the phosphorylation of NFIX has an inhibitory impact on the fusion stage of myogenesis possibly via regulating its DNA binding activity at serine 268 (Figures 4C, 4D, 4E). However, how the phosphorylation events on NFIX affect its regulation of myogenesis (e.g., binding to target genes) requires further investigation.

In summary, our study provides a global perspective on the dynamics and interactions of the signaling networks that underpin myogenesis. Through our prediction framework, which integrates time-course and inhibition phosphoproteome, we were able to dissect activities of closely related kinases and identify their specific substrates. Our findings constitute a valuable resource to aid in the understanding of how upstream signaling pathways regulate downstream transcriptional networks during myogenic differentiation.

### Limitations of the study

As demonstrated in the comparative analysis of our time-resolved proteomic data and the transcriptomes of single cells isolated from *in vivo* skeletal myogenesis during mouse development (Figure 1D), C2C12 myogenic differentiation activates transcriptional and translational responses that closely resemble *in vivo* myogenesis. Although these analyses provide a computational validation of the C2C12 myogenic differentiation system, further study of myogenic signalome using purified primary muscle stem cell populations may further validate the findings made on such *in vitro* systems. The signaling pathways and transcriptional responses identified and characterized from C2C12 myogenic differentiation provide a stepping stone toward understanding primary muscle stem cells and their transition to skeletal muscle *in vivo*.

## STAR★Methods

### Key resources table

**Table:** 

REAGENT or RESOURCE	SOURCE	IDENTIFIER
**Experimental models: Cell lines**		

C2C12 myoblasts	ATCC	RRID: CVCL0188

**Antibodies**		

Anti-FLAG	Sigma	Cat# F1804; RRID:AB_262044
Anti-Myosin	DEVELOPMENTAL STUDIES HYBRIDOMA BANK (DSHB)	Cat# MF20; RRID:AB_2147781

**Deposited data**		

C2C12 time-course phosphoproteome	This paper	PRIDE: PXD028713
C2C12 time-course proteome	This paper	PRIDE: PXD028713
C2C12 inhibition phosphoproteome	This paper	PRIDE: PXD028713
C2C12 inhibition proteome	This paper	PRIDE: PXD028713
C2C12 NFIX overexpression proteome	This paper	PRIDE: PXD028718
Single cell transcriptomes of mouse skeletal myogenesis	(Cao et al., 2019)	GSE119945

**Software and algorithms**		

R version 4.1.1	R Development Core Team, 2016	https://www.R-project.org/
MaxQuant 1.6.12.0	(Cox and Mann, 2008)	http://www.biochem.mpg.de/5111795/maxquant
PhosR 1.2.0	(Kim et al., 2021a)	https://bioconductor.org/packages/PhosR
DirectPA 1.4	(Yang et al., 2014)	https://CRAN.R-project.org/package=directPA
Limma 3.32.2	(Ritchie et al., 2015)	https://bioconductor.org/packages/release/bioc/html/limma.html
ClueR 1.4	(Yang et al., 2015)	https://CRAN.R-project.org/package=ClueR
Minardo-Model	(Kaur et al., 2020)	https://bit.ly/MinardoModel

### Resource availability

#### Lead contact

Further information and requests for reagent and resources may be directed to and will be fulfilled by the Lead Contact, Dr. Pengyi Yang (pengyi.yang@sydney.edu.au).

#### Materials availability

This study did not generate new unique reagents.

### Experimental model and subject details

#### Mice

Female *mus musculus* C2C12 myoblasts were obtained from ATCC (Blau et al., 1985). Cells were cultured in Dulbecco’s Modified Eagle Medium (DMEM) (GIBCO by Life Technologies; # 11995065), supplemented with 10% fetal bovine serum (FBS) (Life Technologies), pyruvate and GlutaMAX (GIBCO by Life Technologies). Cells were kept at 37°C and 5% CO_2_ in a humidified incubator Direct Heat CO_2_ Incubator featuring Oxygen Control (*In Vitro* Technologies).

### Method details

#### Mouse C2C12 myoblast culture and myotube differentiation

Female mouse C2C12 myoblasts (Blau et al., 1985), were grown in Dulbecco’s Modified Eagle Medium (DMEM) (GIBCO by Life Technologies; # 11995065), supplemented with 10% fetal bovine serum (FBS) (Life Technologies), pyruvate and GlutaMAX (GIBCO by Life Technologies). Cells were kept at 37°C and 5% CO_2_ in a humidified incubator Direct Heat CO_2_ Incubator featuring Oxygen Control (*In Vitro* Technologies). Myoblasts between passage 12–15 and at 90% confluence were differentiated into myotubes by replacing 10% FBS with 2% horse serum (HS) (GIBCO by Life Technologies). For MAPK kinase inhibitor studies, myoblasts were treated at the initiation of differentiation with either 0.1% v/v DMSO vehicle control, 1 μM PD0325901 (PD) in 0.1% DMSO or 10 μM SP600125 (SP) in 0.1% DMSO, and cells analysed 2–3 days post-differentiation. For ERK1/2 activation experiments, cells were serum starved in DMEM containing 0.2% bovine serum albumin (BSA) for 2 h followed by stimulation with 100 nM of phorbol 12-myristate 13-acetate (PMA) for 30 min. For over-expression studies, myoblasts in 12-well plates at 60% confluence were transfected with 2 μg DNA, 4 μL of P3000 reagent and 6 μL of Lipofectamne 3000 prepared in reduced-serum Minimal Essential Medium (Opti-MEM) (Life Technologies). Cells were differentiated two days later and analysed at day 2–3 post-differentiation. NFIX cDNA (NM_001371052.1) wild-type and S265/267/268/271/272/273/274/275A was obtained from VectorBuilder and cloned into pcDNA3.1 with N-terminal 3xFLAG-tag. All cell stocks were regularly checked for absence of mycoplasma with the Mycoplasma Detection Kit (Jena Bioscience).

#### Proteome and phosphoproteome sample preparation

Cells were rapidly washed three times with ice-cold PBS and lysed in 4% sodium deoxycholate in 100 mM Tris, pH 8.5 and heated at 95°C for 5min. The samples were tip-probe sonicated and centrifuged at 16,000 x g at 4°C for 15 min, protein quantified by BCA and normalized to 240 μg. Proteins were reduced with 10 mM tris(2-carboxyethyl)phosphine (TCEP) and alkylated with 40 mM 2-chloroacetamide (CAA) at 45°C for 5 min. Samples were digested with sequencing grade trypsin (Sigma #11418025001) and LysC (Wako, Japan) at 1:100 ratio of protease:protein at 37°C for 16h. Ten micrograms of peptide was removed for total proteome analysis and phosphopeptides enriched from the remainder of the sample using the EasyPhos protocol (Humphrey et al., 2018). For NFIX over-expression acquisitions, the sample preparation procedure was identical except only 20 μg of protein was digested and no phosphopeptide enrichment was performed.

#### LC-MS/MS analysis

Peptides were analysed on a Dionex 3500 nanoHPLC, coupled to either an Orbitrap HF-X (temporal analysis of myogenesis) or an Orbitrap Exploris 480 mass spectrometer (MAPK inhibitor and NFIX over-expression analysis) (Thermo Fischer, USA) via electrospray ionization in positive mode with 1.9 kV at 275°C. Separation was achieved on a 40 cm × 75 μm column packed with C18AQ (1.9 μm; Dr Maisch, Germany) over 40 min at a flow rate of 300 nL/min for phosphoproteomics or over 120 min at a flow rate of 300 nL/min for proteomics. The peptides were eluted over a linear gradient of 3–40% Buffer B (Buffer A: 0.1% formic acid; Buffer B: 80% v/v acetonitrile, 0.1%v/v FA) and the column was maintained at 50°C. For the temporal analysis of myogenesis and MAPK inhibitor experiments, the instrument was operated with data-dependent acquisition with MS1 spectra was acquired over the mass range 350–1400 *m/z* (60,000 resolution, 3 × 10^6^ automatic gain control (AGC) and 50 ms maximum injection time) followed by MS/MS analysis of the 10 most abundant ions (phosphoproteomics) or 18 most abundant ions (proteomics) via HCD fragmentation (15,000 resolution, 1 × 10^5^ AGC, 50 ms maximum injection time (phosphoproteomics) or 28 ms maximum injection time (proteomics), 1.6 *m/z* isolation width, 10 ppm dynamic exclusion for 30 s). For NFIX over-expression experiments, the instrument was operated with data-independent acquisition with MS1 spectra was acquired over the mass range 350–950 *m/z* (60,000 resolution, 3 × 10^6^ automatic gain control (AGC) and 50 ms maximum injection time) followed by MS/MS analysis of 38 windows (30,000 resolution, 1 × 10^6^ AGC, auto injection time, 16 m/z isolation width with 1 m/z overlap between windows).

#### Immunostaining and microscopy

Cells were rapidly washed three times with ice-cold PBS and fixed at room temperature for 15 min with 4% paraformaldehyde in PBS. The cells were washed three times with PBS and permeated with 0.1% triton X-100 in PBS at room temperature for 10 min. The cells were washed again three times with PBS and blocked with 3% (w/v) of bovine serum albumin in PBS for 2h at room temperature. Cells were incubated with primary antibody overnight at 4°C. MF20 (1:50; Developmental Studies Hybridoma Bank, University of Iowa, Department of Biology, Iowa City, IA, USA) in 3% BSA/PBS was used to stain myosin heavy chain. Cells were then washed with PBS three times for 5 min each, and incubated for 2 h in the dark, in goat-anti-mouse IgG2b Alexa555 secondary antibody (1:1000, Life Technologies) and DAPI (1:1000) in 3% BSA/PBS. Cells were washed in PBS three times for 5 min each and then imaged on a Zeiss Axiovert 40 CFL inverted microscope using a 10X objective. Four images were taken in each well from pre-defined locations within each quadrant (Caldow et al., 2019).

#### Immunoprecipitation and kinase assay

Forty microliters of Protein-G magnetic beads (Invitrogen) per sample were incubated with 2 μL of anti-FLAG antibody overnight with rotation at 4°C in NP40 Buffer (1% NP40, 10% glycerol, 137 mM NaCl, 25 mM Tris.HCl pH 7.4 containing 10 mM NaF, 10 mM sodium pyrophosphate, 10 mM glycerophosphate, 2 mM sodium orthovanadate and edta-free protease inhibitor cocktail (Roche #11836170001)). The beads were then washed twice with NP40 Buffer prior to use. Cells were rapidly washed three times with PBS and lysed in NP40 Buffer (1% NP40, 10% glycerol, 137 mM NaCl, 25 mM Tris.HCl pH 7.4 containing 10 mM NaF, 10 mM sodium pyrophosphate, 10 mM glycerophosphate, 2 mM sodium orthovanadate and edta-free protease inhibitor cocktail (Roche #11836170001)). The lysate was passed through a 22-guage needle ten times followed by a 27-guage needle three times and centrifuged at 16,000 x g for 10 min at 4°C. Protein was quantified by BCA and 2 mg of protein incubated with the pre-complexed Protein-G:anti-FLAG antibody beads for 2h at 4°C. The beads were washed three times with NP40 Buffer followed by a single wash with Tris-buffered saline. FLAG-tagged protein was competitively eluted off the beads with 25 μg of 3XFLAG peptide (Sigma # F4799) in 30 μL of Kinase Buffer (25mM Tris, 400uM ATP, 10mM MgCl_2_). The elution was divided into two equal aliquots and treated with either 7.5 μL of MillQ or 750 ng / 7.5 μL of active ERK1 (Sigma #E7407) and incubated at 30°C for 1 h. The reactions were quenched with Lamelli Buffer, heated at 65°C for 10 min and separated by SDS-PAGE. The gels were stained overnight at room temperature with Sypro-Ruby (Sigma #S4942) and bands excised on a ChemiDoc (Bio-Rad). Gels bands were wash three times with 50% acetonitrile in 50 mM Tris pH 7.5 and reduced with 10 mM TCEP and alkylated with 40 mM CAA at 45°C for 5 min. Proteins were digested with 14 ng/μL sequencing grade trypsin (Sigma #11418025001) in 100 mM Tris pH 7.5 at 37°C for 16h. Peptides were acidified to a final concentration of 1% trifluoroacetic acid and purified by SBD-RPS microcolumns as described above in the EasyPhos protocol.

#### Phosphoproteomics data analysis

Raw MS data from phosphoproteome and proteome analysis were processed using MaxQuant (version 1.6.12.0) (Cox and Mann, 2008) for phosphosite identification and quantification against mouse UniProt Database (August, 2019 release; 63,738 entries). Data was analysed with default parameters including first search precursor mass tolerance of 20 ppm followed by recalibration and second search set to 7 ppm with fragment ion tolerances set to 0.02 Da. The data was searched with Cys carbamidomethylation set as a fixed modification, and Met oxidation and Ser, Thr and Tyr phosphorylation set as variable modification. All data was filtered to 1% FDR and the PSM and protein level with Match Between Runs enabled and MaxLFQ quantification (Cox et al., 2014). Reverse and containment matches were removed from the MaxQuant output and quantification of phosphorylation level (LFQ intensity values) of each site at the four profiled time points was log-transformed (base 2) before further data preprocessing including filtering, imputation, and normalisation.

For differentiation experiments, phosphosites with at least four quantified values out of all 16 samples and three quantified values out of the four biological replicates in one or more time points were retained. Missing values in the retained phosphosites were imputed first by a site- and condition-specific imputation method, where for a phosphosite that contains missing values at a time point, if more than two samples were quantified in that time point, the missing values were imputed based on these quantified values for that phosphosite in that time point; and then by a random-tail imputation method (Kim et al., 2021b). This strategy has been found to be effective since for a phosphosite it takes into account the quantified value when such information is available or otherwise simulates the low detection boundary when no quantification is available (Yang et al., 2019). The imputed data were converted to ratios relative to 0 h and normalised using Combat (Johnson et al., 2007) for removing batch effect and then phosphoRUV using PhosR (Kim et al., 2021a) for removal of additional unwanted variation. Finally, the normalised phosphoproteomic data at 24 h and day 5 were corrected by the total proteome data at the corresponding time points to account for protein changes. For inhibition experiments, phosphosites first were filtered for at least three quantified values out of 12 samples and three quantified values out of four biological replicates in one or more conditions. Missing values were imputed similarly as was performed in the differentiation experiments and imputed data were converted to ratios relative to control samples and further normalised by the total proteome from the corresponding conditions to account for protein changes.

#### Proteomics data analysis

Raw MS data of proteome analyses from differentiation and inhibition experiments were processed using MaxQuant (version 1.6.12.0) for protein identification against mouse UniProt database (August, 2019 release; 63,738 entries). Data was analysed with default parameters as described above without the inclusion of phosphorylation as a variable modification. Reverse and containment matches were removed and the LFQ intensity quantifications of the proteins were log-transformed (base 2) prior to subsequent analyses. For differentiation experiments, proteins were filtered requiring at least two out of four biological replicates to be quantified in at least two adjacent time points across the time-course. Missing values in the proteins that passed the filtering were imputed first by a site- and condition-specific imputation method, where for a protein that contains missing values at a time point, if more than two samples were quantified at that time point, the missing values were imputed based on the quantified values for that protein at that time point. The remaining missing values were imputed by a random-tail imputation method similar to the above phosphoproteomics data analysis but with the parameters recommended in (Beck et al., 2015). The imputed data were next converted to ratios relative to 0 h and normalised using Combat for removing batch effect and RUV for removing additional unwanted variation using stably expressed genes as negative controls (Lin et al., 2019). For inhibition experiments, we filtered proteins retaining those that have at least four quantified values out of fifteen samples and three quantified values out of four biological replicates. Missing values were imputed similarly as was performed in the differentiation experiments and the imputed data were next converted to ratios relative to control and normalised using RUV.

For NFIX mutagenesis experiments, raw MS data were processed using Spectronaut (version 15.0.210615.50606) and searched against mouse UniProt database (August, 2019 release; 63,738 entries) using default settings with precursor, peptide and protein FDR set to 1%. Quantification was performed at the MS2-level using 3–6 fragment ions including automated interference removal (Bruderer et al., 2015). All other quantification settings were set to default except imputation was disabled and filtered based solely on Q-value. Data were first log2 transformed and missing values were imputed using random-tail imputation method as described above. The imputed data were then converted to ratio relative to GFP samples and normalised using RUV with stably expressed genes.

#### Correlation analysis with single cell transcriptomes from *in vivo* skeletal myogenesis

For comparison of the C2C12 time-course proteome with the single cell transcriptomes generated from *in vivo* skeletal myogenesis during mouse development (E9.5-E13.5) (Cao et al., 2019), we extracted the myotubes by using marker genes Myf5, Myod and Myog (Myf5 + or Myod+ and Myog−). Next, we filtered genes that have > 95% of zeros and selected the top 100 highly variable genes (HVGs). Lastly, Pearson’s correlation was performed between the transcriptome of each myotube from the mouse development data and the C2C12 proteome of each time point using HVGs.

#### Kinase activity inference and kinase-substrate prediction

Kinase activities at the time points of 30 min, 24 h, and day 5 were inferred based on the changes of phosphorylation (relatively to myoblasts at the 0 time point) of their known substrates using the KinasePA package (Yang et al., 2016) and the PhosphoSitePlus annotation database (Hornbeck et al., 2012). Putative substrates of kinases were predicted using the ‘kinaseSubstrateScore’ function in PhosR package (Kim et al., 2021a), a machine learning approach that learns from the combined features of phosphorylation profiles and sequence motifs that each kinase recognise. The prediction scores were subsequently used for constructing signalome networks, using the ‘Signalomes’ function in PhosR, based on the sets of shared and unique proteins each kinase phosphorylates, and the results from this analysis were presented as a chord diagram.

To identify MAPK1/3 and MAPK8/9 specific substrates, predictions for both these two groups of kinases were filtered for those with prediction scores greater than 0.7. Predictions that passed the filtering were then classified to two groups based on their inhibition profiles to those that are more inhibited by PD than SP (MAPK1/3-specific) and those in reverse (MAPK8/9-specific).

#### Temporal clustering and ordering of time course proteome during C2C12 differentiation

For clustering analysis, proteome data from time-course differentiation of C2C12 myoblasts were first used to identify dynamically regulated proteins (see Quantification and statistical analysis section) (Table S8). For the proteins that are dynamically regulated during the myogenesis, protein fold changes (relative to 0) were first z-score transformed across time points and then clustered based on their temporal profiles. The R package ClueR (Yang et al., 2015) was used for determining the optimal clustering based on the Reactome pathway database (Fabregat et al., 2018) and Fisher’s exact test was used to identify enriched pathways in each cluster. Lastly, the Minardo R package (Kaur et al., 2020) was used for determining the order of the pathway events based on the temporal profile of each cluster.

#### Image analyses of morphologic changes

Images of myotubes generated from the inhibition experiment under normal differentiation condition (control) or the addition of PD (MAPK1/3 inhibitor) and SP (MAPK8/9 inhibitor) were analysed for identifying morphologic changes under kinase inhibitions compared to controls. Cells of each condition were cultured in three separate wells and microscopic images of four fields in each well were randomly captured for morphology analyses. Features include nucleus counts, MHC + cell counts, and myotube counts were measured using ImageJ software (NIH, Bethesda, MD) (Abràmoff et al., 2004). The total number of nuclei, MHC + cells and myotube and their standard deviations of each condition were calculated from the microscopic images of three separate wells. Differentiation index was calculated by dividing the number of nuclei within MHC + cells by the total number of nuclei. The percentage of MHC + cells was calculated by dividing the number of MHC + cells by the total number of nuclei. Fusion index was calculated by dividing the number of nuclei within multinucleated (≥4) myofibers by the total number of nuclei (Bello et al., 2009).

### Quantification and statistical analysis

#### Dynamically regulated proteins

Dynamically regulated proteins across time points during myogenic differentiation of C2C12 cells were defined as having absolute log2 fold changes (relative to 0h) greater than 1 in at least two time points and an FDR adjusted p-values of 0.05 or smaller from an ANOVA test across all profiled time points.

#### Inhibition specificity analysis

Kinases and pathways inhibited by PD and SP were analysed integratively using KinasePA (Yang et al., 2016) for the phosphoproteomic data and directPA for the proteomic data (Yang et al., 2014). Statistical significance of phosphosites and proteins on inhibition specific to PD or SP treatments or co-inhibition by both inhibitors were analysed, and the enrichment of their corresponding kinases and pathways were tested.

#### Differentially expressed proteins

Differentially expressed (DE) proteins from NFIX overexpression analysis were identified using limma R package (Ritchie et al., 2015). In particular, NFIX-WT and NFIX-mutant overexpression samples were first compared to GFP overexpression samples and then the normalised ratios of NFIX-WT were compared to those from NFIX-mutant for identifying DE proteins using the moderated *t*-test in limma. Proteins that have an absolute log2 fold change of 1 and an FDR-adjusted p-value < 0.05 were called as DE proteins.

## Data Availability

The myogenesis differentiation and inhibitions phosphoproteomics and proteomics data generated in this study are deposited to the ProteomeXchange Consortium (http://proteomecentral.proteomexchange.org/cgi/GetDataset) via the PRIDE (Perez-Riverol et al., 2019) partner repository under PRIDE:PXD028713. The NFIX over-expression data can be accessed under PRIDE:PXD028718. This paper does not report original code. Any additional information required to reanalyze the data reported in this paper is available from the lead contact upon request.

## Supplemental information

Document S1. Figures S1–S4

Table S1. Time-course phosphoproteome, related to Figure 1

Table S2. Time-course proteome, related to Figure 1

Table S3. Inhibition phosphoproteome, related to Figure 3

Table S4. Inhibition proteome, related to Figure 3

Table S5. Direction analysis of inhibition data, related to Figure 3

Table S6. NFIX overexpression proteome, related to Figure 4

Table S7. Differential expression analysis of NFIX overexpression, related to Figure 4

Table S8. Dynamically regulated proteins of time-course proteome, related to Figure 2
