# Supplementary material for: Time-resolved phosphoproteome and proteome analysis reveals kinase signaling on master transcription factors during myogenesis
Source: iScience. 2022 May 30;25(6):104489. doi: 10.1016/j.isci.2022.104489 (PMC9198430; doi:10.1016/j.isci.2022.104489)
Supplement: Document S1. Figures S1–S4 [file mmc1.pdf]

iScience, Volume *25*

Supplemental information

Time-resolved phosphoproteome and proteome analysis reveals kinase signaling on master transcription factors during myogenesis

Di Xiao, Marissa Caldow, Hani Jieun Kim, Ronnie Blazev, Rene Koopman, Deborah Manandi, Benjamin L. Parker, and Pengyi Yang

Supplementary Figures 1-4

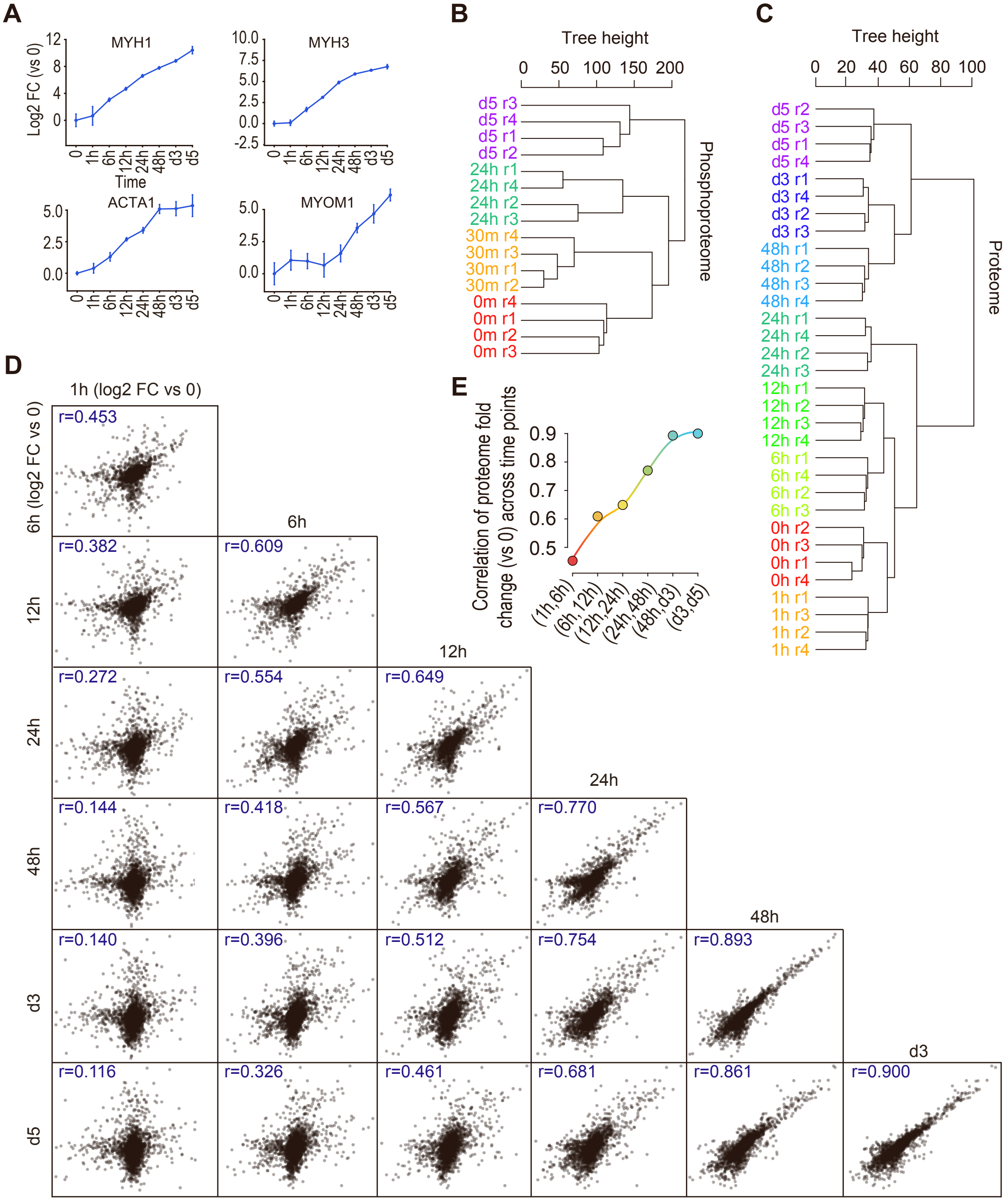

Supplementary Figure 1 (Related to Figure 1). Myogenic Differentiation of C2C12 Murine Myoblasts.

(**A**) Temporal dynamics of proteome abundance (compared to 0) of myogenic markers (MYH1, MYH3, ACTA1, MYOM1) during C2C12 differentiation. Error bars represent SD from the four biological replicates. (**B, C**) Unsupervised hierarchical clustering of the phosphoproteome (**B**) and proteome (**C**) of the four biological replicates at the indicated time points during myogenic differentiation of C2C12. Samples are coloured by their corresponding time points. (**D**) Pairwise scatter plots visualising temporal dynamics of proteome abundance (relative to 0) at any two profiled time points (except 0) during C2C12 differentiation. Pearson's correlation coefficients are calculated for each pairwise comparison. (**E**) Temporal dynamics of correlation (y-axis) between proteome fold-changes at each pair of adjacent time points (compared to 0) across the differentiation process.

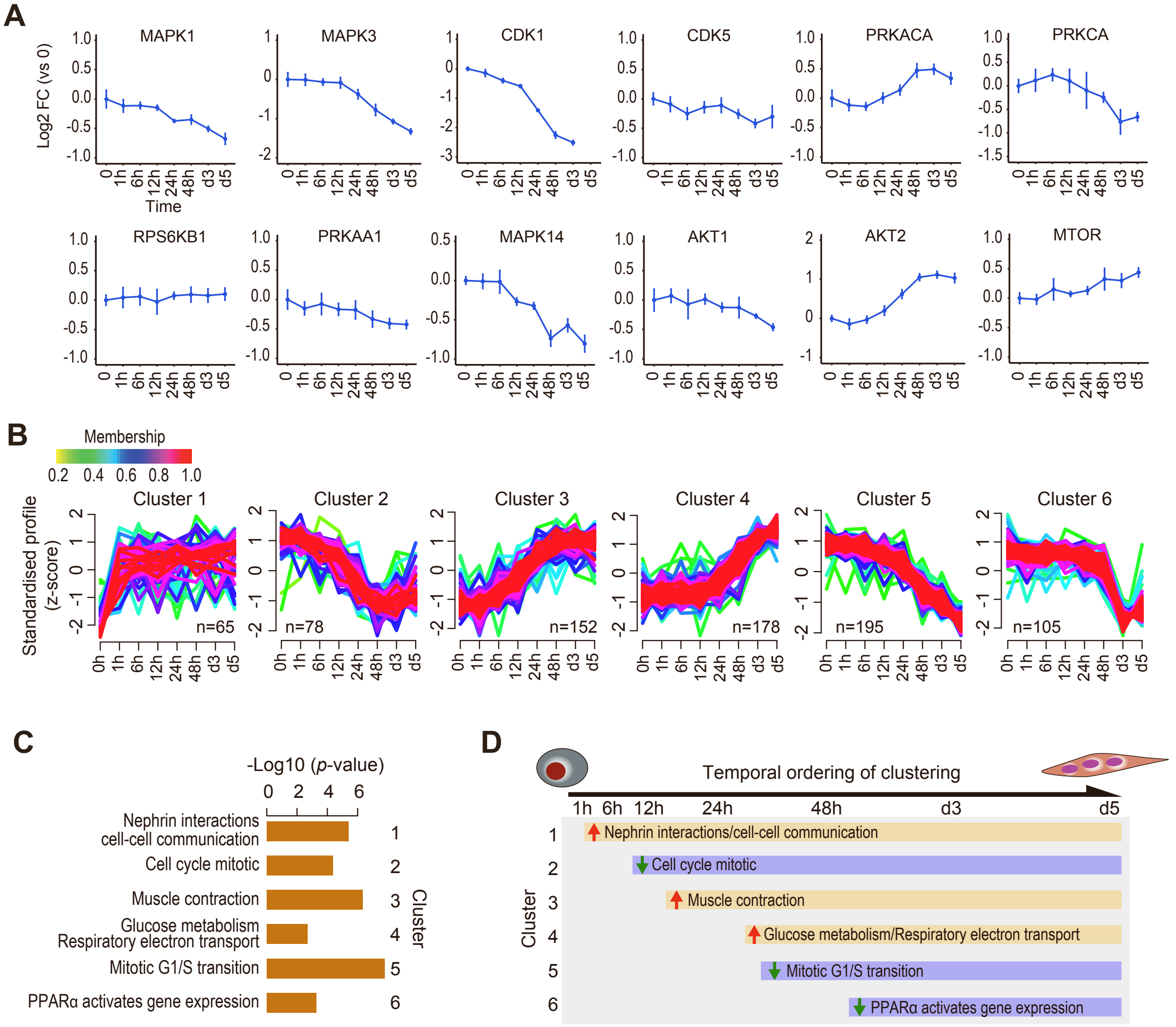

Supplementary Figure 2 (Related to Figure 2). Characterisation of Key Kinases and Global Translational Dynamics of C2C12 Myogenesis.

(**A**) Temporal dynamics of protein abundance (compared to 0) of select kinases during C2C12 myogenic differentiation. Error bars represent SD from the four biological replicates. (**B**) Temporal clusters visualising differentially regulated proteins (see STAR Methods) during C2C12 differentiation. Y-axes are z-score standardise profiles capturing protein dynamics regardless of their magnitude of changes. Number of proteins included in each cluster is noted. (**C**) Bar plot showing the enriched pathways from each of the six temporal clusters in (**B**). (**D**) Temporal ordering of clusters from (**B**). Each pathway is coloured by their global dynamics with yellow representing up-regulation and purple representing down-regulation during myogenesis.

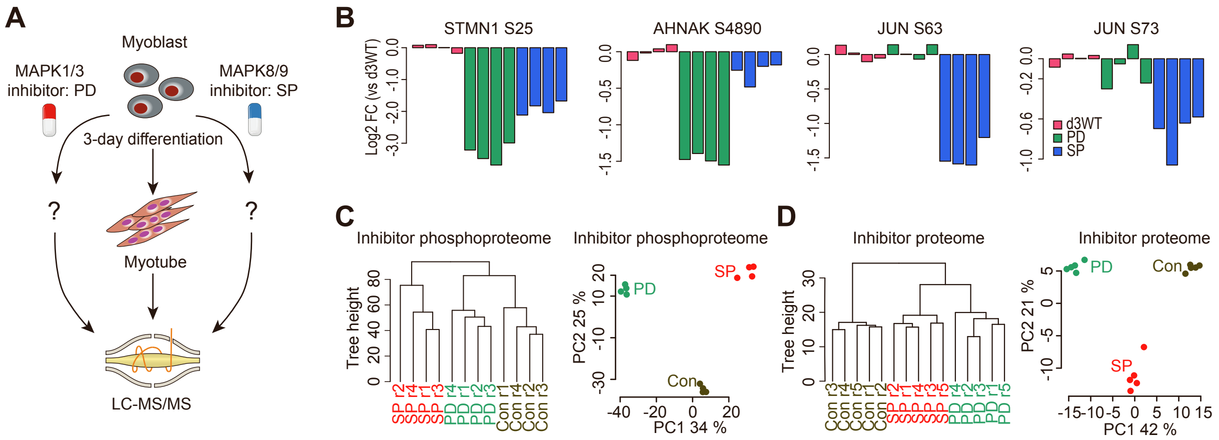

Supplementary Figure 3 (Related to Figure 3). Dissecting MAPK1/3 and MAPK8/9 Specific Effects on C2C12 Myogenesis.

(**A**) Schematic summary of myogenic differentiation of C2C12 without and with MAPK1/3 or MAPK8/9 inhibition. Phosphoproteome and proteome were profiled at day-3 after differentiation induction. (**B**) Phosphorylation profiles of known substrates of MAPK1/3 (AHNAK S4890 and STMN1 S25) and MAPK8/9 (JUN S63 and JUN S73) under PD and SP treatments (compared to control). (**C**) Hierarchical clustering and PCA plot of the phosphoproteomes from the MAPK inhibition experiments. Samples are coloured by experimental conditions. (**D**) Hierarchical clustering and PCA plot of the proteomes from the MAPK inhibition experiments. Samples are coloured by experimental conditions.

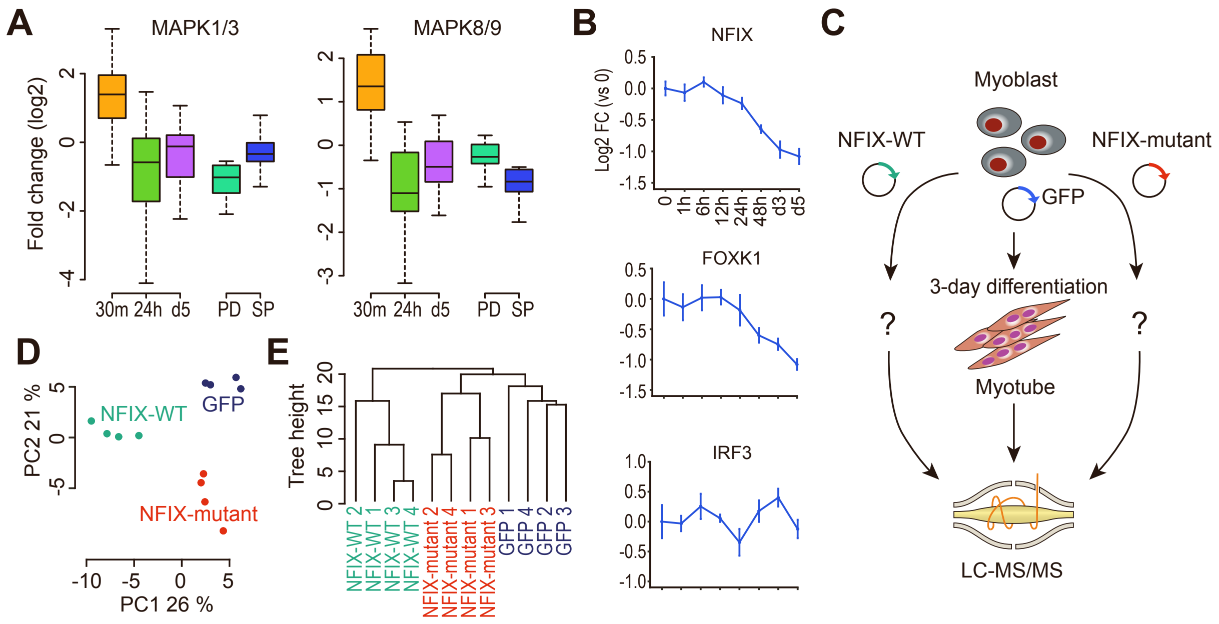

Supplementary Figure 4 (Related to Figure 4). Identification and Validation of NFIX as a Novel Regulator in Myogenesis.

(**A**) Boxplots showing the temporal and inhibition phosphorylation profiles of predicted substrates of MAPK1/3 and MAPK8/9, respectively. (**B**) Temporal dynamics of protein abundance (compared to 0) of predicted TFs of MAPK1/3 (Figure 4A) during C2C12 myogenic differentiation. Error bars represent SD from the four biological replicates. (**C**) Schematic summary of myogenic differentiation of C2C12 with overexpression of GFP, wide-type NFIX (NFIX-WT) or phospho-dead mutant of NFIX (NFIX-mutant). Proteomes were profiled at day-3 after differentiation induction. (**D, E**) PCA plot (**D**) and hierarchical clustering (**E**) of the proteomes profiled from the NFIX overexpression experiments. C2C12 myoblasts overexpressing GFP, NFIX-WT or NFIX-mutant were differentiated and profiled at day-3. Colours correspond to overexpression conditions.
